# The Role of Illness-Related Beliefs in Depressive, Anxiety, and Anger Symptoms: An On-line Survey in Women With Hypothyroidism

**DOI:** 10.3389/fpsyt.2021.614361

**Published:** 2021-04-22

**Authors:** Daniel Pankowski, Kinga Wytrychiewicz-Pankowska, Konrad Janowski, Ewa Pisula, Magdalena Walicka

**Affiliations:** ^1^Department of Psychology, University of Warsaw, Warsaw, Poland; ^2^Department of Psychology, University of Economics and Human Sciences in Warsaw, Warsaw, Poland; ^3^Department of Internal Diseases, Endocrinology, and Diabetology, Central Clinical Hospital of the Ministry of the Interior and Administration in Warsaw, Warsaw, Poland; ^4^Department of Human Epigenetics, Mossakowski Medical Research Institute Polish Academy of Sciences, Warsaw, Poland

**Keywords:** hypothyroidism, cognitive representation of illness, depressive symptoms, anxiety, anger

## Abstract

Hypothyroidism may affect 3–8.5% of the population and is a growing global health problem.

**Objective:** The aim of the current study was to assess the relationships between cognitive representations of this illness and the severity of symptoms of depression, anxiety, and anger in women who suffer from hypothyroidism.

**Methods:** The study used a cross-sectional design with on-line recruitment and measurements. A total of 354 women took part in the study and completed the following questionnaires: a 5-point self-rating scale that measures the three major symptoms of hypothyroidism, the Illness-Related Beliefs Questionnaire, the Hospital Anxiety and Depression Scale—Modified (HADS-M), and a clinical and sociodemographic data questionnaire.

**Results:** The study found a relationship between the severity of emotional distress symptoms and illness-related beliefs. These beliefs were correlated with depressive symptoms, anxiety, and anger regardless of age, education, hormone levels or time since the diagnosis. In addition, the results of regression analyses, both hierarchical and stepwise, indicated that beliefs about the disease explained relatively high levels of the outcome variables (about 30% of the variance of depressive and anxiety symptoms and 16% of anger) as measured by HADS-M.

**Conclusions:** Psychological factors seem to play an important role in the development of symptoms of depression, anxiety, and anger in patients with hypothyroidism. Psychosocial interventions targeting personal beliefs about the nature of the disease and its social aspects may be an effective way to reduce emotional distress symptoms.

## Introduction

Hypothyroidism is a common clinical syndrome caused by an increase in concentration of Thyroid-Stimulating Hormone (TSH) above the normal reference range and a decline of the level of free thyroxine (fT4). Subclinical hypothyroidism is diagnosed when TSH levels are above the standard range but levels of fT4 fall within the reference range for the population (1). The symptoms of hypothyroidism include fatigue, sensitivity to cold, dry skin, sleepiness, weight gain, constipation, and psychological disorders (2). Overt hypothyroidism is treated with thyroid hormones [Levothyroxine; (3)] and subclinical hypothyroidism also often needs such supplementation. Hypothyroidism mainly affects people aged 30–60 and women constitute about 80% of patients. In the United States' National Health and Nutrition Examination Survey (NHANES III), overt hypothyroidism was detected in 0.5% of the population while its subclinical form was found in 0.7% of the general population (4). Other studies have shown that hypothyroidism may affect 3–8.5% of the population (5) and should be regarded as a growing, global health problem which can significantly adversely affect quality of life (6–8).

It is believed that thyroid diseases may be associated with deterioration of psychological functioning (9). Patients suffering from hypothyroidism often report the occurrence of emotional distress (ED), typically in the form of depression, anxiety and anger (10, 11). Boswell et al. (12) showed that depression occurs in nearly 50% of cases of hypothyroidism. In another study, 60% of patients with hypothyroidism reported some degree of depressive symptoms and 63% reported some degree of anxiety symptoms (13). Additionally, feelings of anger have been found to be associated with chronic diseases (14) and, in patients with hypothyroidism, levels of anger have been found to be related to treatment effectiveness (11). Despite the evidence of a link between thyroid dysfunctions and anxiety symptoms and mood (15), there is still disagreement regarding the paths linking thyroid diseases with ED (16). Some studies have managed to demonstrate the existence of such a link and showed a reduction in the incidence of depressive symptoms during Levothyroxine treatment (17), while other studies indicated a lack of relationship between thyroid functions and depressive and anxiety symptoms (18). One of the studies showed that, regardless of the severity of hypothyroidism, the incidence of depressive symptoms was much higher in people who had known about their diagnosis for a long time in comparison to those diagnosed recently (19). Overall, it seems there is sufficient evidence in the literature supporting a relationship between thyroid dysfunction and ED; however, the mechanisms leading to ED in hyperthyroidism are not fully understood.

In the current study, we would like to put forward and test the claim that this association is mediated by the cognitive factors involved in the psychological adaptation processes triggered by chronic medical conditions (20).

Illness representation is one of the cognitive factors related to how an individual perceives their disease and its consequences. This concept was introduced by Leventhal et al. (21, 22) in what is known as the Common Sense Model of illness. It describes a set of beliefs covering five key aspects of a disease (23): (1) the identity component—patients' ideas about the label of their condition, its nature (i.e., associated symptoms), and the links between them; (2) the causal component—the patient's beliefs about the likely cause or causes of the illness; (3) the time-line component—beliefs concerning the likely duration of the health problems (acute/short-lasting, chronic, or cyclical/episodic); (4) the consequences component—includes the individual's beliefs about the severity of the illness and its likely impact on their physical, social, and psychological functioning; and (5) the cure component—the extent to which the patient believes their condition can be cured or controlled.

Although illness representation is most commonly operationalized as a structure consisting of these five categories of beliefs (21, 22), the actual structure of illness-related beliefs is most probably much more complex. It seems that some other important categories of beliefs could also be included in the structure of illness representation. For instance, Leventhal's model seems to omit beliefs related to the social aspect of illness, which is of key importance for patients, especially with visible symptoms, such as with skin diseases (24). It is obvious that diseases differ in the degree of social stigma attached to them (25) and even patients with the same illness may vary in their beliefs about how stigmatizing or embarrassing their illness is (26). Studies also demonstrate that a patient's level and accuracy of knowledge about their illness may be relevant to their adjustment to a chronic illness, as expressed in their health-related behaviors, mood, etc. (27), and this is obviously mediated by their subjective beliefs about how extensive and accurate their knowledge of the disease (28). The patient's beliefs about the severity of their condition in comparison to that of other patients with the same illness may also affect adaptational outcomes (29). Therefore, in our study, we decided to also take into account categories of illness-related beliefs of this type, even though they were not included in the original five-component model of illness representation. This reflects our view that this extended model of illness representation could be a better operationalization of this concept.

Previous studies have shown that illness representation plays a critical role in the process of adaptation to a chronic disease (30). It has been emphasized that illness representation may determine a patient's subsequent cognitive and emotional responses to illness and its symptoms (21) and is related to ED—in particular, levels of depression (31) and anxiety (32). Research has shown that illness representation can also be crucial for other areas of functioning, such as health-related behavior and the outcomes of interventions aimed at preventing diseases (33).

The aim of the current study was to assess the associations between cognitive representations of illness and ED in the form of depressive, anxiety, and anger symptoms in women suffering from hypothyroidism. On the theoretical level, we decided to expand the original five-component model of illness representation (21) by including several other illness-related beliefs which may prove important when accounting for adaptational outcomes in patients with a chronic illness. In addition, when analyzing the role of illness representation for ED, we undertook to control for the potentially confounding effects of clinical and sociodemographic variables.

## Materials and Methods

### Measurement Instruments

Sociodemographic Data Sheet - the respondents were asked about their age (in years); marital status (married/in an informal relationship/single) and level of education (primary school/vocational school/high school/higher education). Whenever categorical variables were inappropriate for a statistical analysis (i.e., regression analysis and partial correlation analysis), the marital status and education level variables were dichotomized using dummy-coding.

Hypothyroidism Symptoms Severity –a 5-point self-rating scale was used to measure the three major symptoms of hypothyroidism. Participants responded to questions about the severity of feeling cold, swelling of hands and/or feet, and dry skin over the 4 weeks prior to the study on a scale with responses anchored as: “not at all,” “a little,” “average,” “quite a lot,” “very much.”

The Illness-Related Beliefs Questionnaire (IRBQ) was developed by Janowski and Pankowski to assess the intensity of a patient's personal beliefs pertaining to key aspects of their chronic disease. It consists of 13 beliefs covering five categories of beliefs previously described in the literature [see: (34)] and several additional beliefs (such as those regarding self-knowledge, comparisons to other patients, social stigma, etc.). Each belief is expressed on a continuum ranging from one extreme to another, for example: “My illness will last for a short time — My illness will last for a long time.” Respondents are requested to locate their own personal belief on this continuum using a 1–10 response scale. The contents of the 13 beliefs measured by the IRBQ are presented in the **Appendix** to this article. For the purposes of this study, the instruction was adjusted to the health problem experienced by the patients (i.e., hypothyroidism). The psychometric properties of this tool are the subject of a separate publication, which is currently in preparation.

The Hospital Anxiety and Depression Scale—Modified (HADS-M) was used to examine the severity of the depressive and anxiety symptoms most common in health-service settings. The original HADS was developed by Zigmond and Snaith (35). The Polish version of the HADS-M was used (36, 37) in the present study. The Polish adaptation was modified by inserting two additional items about outbursts of anger (Item 15) and feeling nervous and losing one's temper (Item 16), which together constitute a subscale labeled “anger.” In this version, the tool is a 16-item self-report scale which measures the presence of symptoms of anxiety (seven items; Cronbach's alpha = 0.82), depression (seven items; Cronbach's alpha = 0.87), and anger (two items; Cronbach's alpha = 0.84) over the past 4 weeks. Each item is scored on a scale from 0 to 3.

The Clinical Course of Hypothyroidism Questionnaire: a structured questionnaire was used in which participants provided information on: time since their hypothyroidism was diagnosed (in years), TSH, fT3 (free triiodothyronine) and fT4 levels with units from tests performed not earlier than 30 days before participating in the study, and the treatment used with L-thyroxine dose.

### Procedure and Participants

The study was carried out on-line with a Polish-speaking sample. The questionnaires were prepared using Google forms and then advertised on online Facebook forums for people suffering from thyroid diseases. In the case of questionnaires, the respondents had to answer all the questions in order to proceed to the further part of the study. Participation was anonymous and no remuneration was provided. Due to the fact that there was no direct access to the subjects, e.g., in medical clinics, the focus was on people undergoing treatment and with normalized hormone levels. Thanks to this, it was possible to obtain a coherent group.

The inclusion criteria were women aged > 18 years old who declared that they have hypothyroidism and are undergoing L-thyroxine treatment. Due to the fact that hypothyroidism is 10 times more common in women than in men (38), it was decided before recruitment that the study would be addressed only to women in order to ensure the greatest possible cohesion of the group.

A total of 24 people were excluded from the analysis due to taking medicines used for hyperthyroidism or failure to fill-in information regarding the treatment. Thyroid hormone levels and other clinical data were self-reported by participants.

### Ethical Approval

This study was conducted in accordance with the guidelines of the Declaration of Helsinki. The approval of the local Bioethical Commission was obtained for this study and all participants gave informed consent prior to their participation in the study.

### Analysis

In the first step, partial correlations were computed between ED (depressive, anxiety and anger symptoms) and clinical variables (severity of symptoms, TSH, L-thyroxine dose, time since diagnosis, age at diagnosis) while controlling for sociodemographic variables (age, marital status, and education).

In the next step, partial correlations were calculated between ED and illness-related beliefs with both sociodemographic and clinical variables entered as controls.

In order to assess the cumulative contribution of separate groups of variables to the variance of ED, a series of hierarchical regression analyses were carried out. In regression analyses, scores for depressive symptoms, anxiety, and anger were introduced individually as dependent variables and the following variables were entered as independent variables in blocks:

- sociodemographic data (age, marital status, and education);
- clinical data (severity of symptoms, TSH, L-thyroxine dose, time since diagnosis, age at which diagnosis was made);
- illness-related beliefs.

Individual predictors of ED were identified by conducting a series of stepwise regression analyses. Scores for symptoms of depression, anxiety and anger were introduced as dependent variables, while all independent variables were introduced in one block. All analyses were conducted using IBM SPSS version 25.

## Results

### Descriptive Statistics

The study sample consisted of 354 women with a mean age of 33.80 (SD = 9.82) years. Age ranged from 18 to 70 years. About half of the respondents (50.8%) were married, 18.6% were single, and 30.5% were in an informal partnership. About a quarter of patients (24.3%) had secondary education, 73.2% had higher education, 2.3% had vocational education, and 0.3% had primary education.

The mean time elapsed since diagnosis was 5.15 (SD = 4.64) years, and this ranged from 0 (this year) to 24 years. The TSH index ranged from 0.2 to 9 μIU/mL (M = 2.44, SD = 1.91). The fT3 values ranged from 0.13 to 1.14 ng/dL (M = 0.32, SD = 0.14) and fT4 values ranged from 0.32 to 2.44 ng/dL (M = 1.27, SD = 0.32).

Descriptive Statistics for the IRBQ and HADS-M are presented in Table 1.

**Table 1:** IRBQ and HADS-M descriptive statistics.

**Variables**	**M**	**SD**	**Range**
My illness will last for a very long time.	9.18	1.89	1–10
This illness will have a very significant impact on my life.	7.85	2.39	1–10
My condition will worsen.	5.93	2.41	1–10
The symptoms of my illness are very visible to others.	5.23	2.89	1–10
I cannot predict the course of my illness at all.	6.87	2.44	1–10
I know almost nothing about my illness.	3.95	2.26	1–10
Things I have done caused me to become ill.	3.43	2.67	1–10
The things I do have no effect on the course of my illness.	3.98	2.53	1–10
Medical staff cannot influence the course of my illness.	5.45	2.73	1–10
The treatment I get does not help me at all.	4.98	2.18	1–10
In general, others would regard me negatively due to the fact I have this illness.	4.67	2.43	1–10
I believe my illness is embarrassing.	2.60	2.46	1–10
Compared to other people who have this illness, my symptoms are very severe.	4.34	2.31	1–10
HADS—Anxiety	10.23	4.40	0–21
HADS—Depression	6.71	4.33	0–19
HADS—Anger	4.06	1.67	0–6
Feeling cold	3.51	1.33	1-5
Swelling of hands and/or feet	2.54	1.34	1-5
Dry skin	3.86	1.10	1-5

Due to the lack of cut-off points for the Polish version of the HADS-M, we used the values proposed by Snaith and Zigmond (39) to investigate the prevalence of clinically important anxiety and depressive symptoms. We found that 19.8% (*n* = 70) of women had borderline depressive symptoms (8–10 points), 14.97% (*n* = 53) had mild depressive symptoms (11–14 points), and 5.09% (*n* = 18) had severe depressive symptoms (≥15 points). Also, 22.6% (*n* = 80) of participants had borderline anxiety symptoms (8–10 points) and 48.3% (*n* = 171) had mild anxiety symptoms (11, 13–15).

### Correlates of Anxiety, Depressive Symptoms, and Anger

When controlling for sociodemographic variables, statistically significant partial correlations were found between ED symptoms and subjective clinical symptoms of hypothyroidism (feeling cold, swelling of hands and/or feet, and dryness of the skin). The outcome variables did not correlate significantly with the remaining clinical variables: levels of thyroid hormones, L-thyroxine doses, time since diagnosis, or age at diagnosis (Table 2).

**Table 2:** Partial correlations between the ED (depressive symptoms, anxiety, anger) and clinical variables (controlled by sociodemographic variables).

	**Anxiety**	**Depression**	**Anger**
Cold intolerance	0.21^**^	0.17^*^	0.20^**^
Swelling of hands and/or feet	0.26^***^	0.19^*^	0.19^**^
Dryness of the skin	0.17^*^	0.02	0.13
TSH (μIU/mL)	−0.03	0.03	0.03
fT3 ng/dL	−10	−0.05	−0.12
fT4 ng/dL	−0.11	−0.12	−0.04
L-thyroxine doses	−0.07	−0.07	−0.13
Time since diagnosis	−0.08	−0.09	−0.06
Age at diagnosis	0.08	0.09	0.06

* p < 0.05;

** p < 0.01;

*** *p < 0.001*.

Analysis of the data showed that, after controlling for sociodemographic variables, depressive symptoms, anxiety, and anger were related only to subjectively assessed symptoms (i.e., cold intolerance, swelling of hands and/or feet, and dryness of the skin). Statistically significant correlation coefficients ranged from 0.17 to 0.26. There was no association between ED and variables the level of hormones, L-thyroxine treatment doses, or time elapsed since the diagnosis.

Partial correlations between the outcome variables (depressive symptoms, anxiety and anger) and illness-related beliefs are presented in Table 3. In this analysis, both sociodemographic and clinical variables were entered as controlled variables.

**Table 3:** Partial correlations between the outcome variables (depressive symptoms, anxiety, anger) and illness-related beliefs (controlling for both sociodemographic and clinical variables).

	**Anxiety**	**Depression**	**Anger**
Illness will last for a very long time	0.00	0.01	0.00
Illness will have very significant impact on life	0.11	0.14	0.23^**^
Condition will worsen	0.21^**^	0.19^**^	0.17^*^
Symptoms very visible to others	0.36^***^	0.33^***^	0.24^**^
Cannot predict course	0.22^**^	0.20^**^	0.20^**^
I know almost nothing about my illness	0.34^***^	0.25^***^	0.13
Things I have done caused me to become ill	0.13	0.08	−0.03
Things I do have no effect on my illness	0.13	0.07	0.01
Medical staff cannot influence the course of my illness	0.28^***^	0.26^***^	0.25^***^
The treatment I get does not help me at all	0.34^***^	0.36^***^	0.23^**^
Others would regard me negatively due to the fact I have this illness	0.25^***^	0.36^***^	0.09
My illness is embarrassing	0.32^***^	0.38^***^	0.16^*^
Compared to other people who have this illness, my symptoms are very severe	0.40^***^	0.36^***^	0.21^**^

* p < 0.05;

** p < 0.01;

*** *p < 0.001*.

All three outcome variables showed statistically significant associations with the majority of illness-related beliefs, even after controlling for the effects of sociodemographic and clinical variables (Table 3). In general, depressive symptoms, anxiety, and anger became more severe with increasing intensity of negatively oriented illness-related beliefs. Statistically significant correlation coefficients ranged from 0.16 to 0.4. A similar pattern of correlations with illness-related beliefs can be noticed for depressive symptoms and anxiety. In the case of anger, the correlation coefficients were generally lower. The strongest correlations were observed for beliefs regarding the social aspects of the disease such as the belief about the embarrassing nature of illness or about visibility of symptoms to other people.

### Predictors of Anxiety, Depressive Symptoms, and Anger

Table 4 presents the results of a series of hierarchical regression analyses computed separately for each ED variable (depressive symptoms, anxiety, and anger). Out of the three groups of independent variables, only illness-related beliefs were found to be significant predictors of variance in depressive symptoms. Both illness-related beliefs and clinical variables made a statistically significant contribution to explaining the variance in anxiety (when IRBs were entered as last). All three groups of independent variables (sociodemographic, clinical, and illness-related beliefs) were statistically significant predictors of anger.

**Table 4:** The role of sociodemographic and clinical variables and illness-related beliefs in explaining the variance of ED symptoms—hierarchical regression analysis.

**Hierarchical regression model for:**	**Independent variables (entered in blocks)**	***R***	***R*^**2**^**	**Adjusted *R*^**2**^**	***R*^**2**^ change**	**change in F**	**Significance of change in F**
Depressive symptoms	Sociodemographic variables	0.144	0.021	0.005	0.021	1.299	0.276
	Clinical variables	0.287	0.083	0.036	0.062	2.015	0.066
	Illness-related beliefs	0.625	0.391	0.310	0.308	6.466	0.000
Anxiety	Sociodemographic variables	0.116	0.013	−0.003	0.013	0.842	0.473
	Clinical variables	0.342	0.117	0.073	0.104	3.497	0.003
	Illness-related beliefs	0.654	0.428	0.353	0.311	6.956	0.000
Anger	Sociodemographic variables	0.211	0.045	0.029	0.045	2.873	0.038
	Clinical variables	0.341	0.116	0.072	0.072	2.425	0.028
	Illness-related Beliefs	0.520	0.271	0.174	0.154	2.700	0.002

To analyze the specific (non-redundant) contribution of clinical variables to the variance in the outcome variables, clinical variables were entered into regression models as the last block. This analysis showed that the specific (non-redundant) contribution of clinical variables was 3.1% (*p* = 0.216) in accounting for the variance of depressive symptoms, 3.9% (*p* = 0.089) for anxiety, and 3.6% (*p* = 0.236) for anger.

The results of stepwise regression analyses are presented in Table 5.

**Table 5:** Predictors of the severity of ED symptoms in the group of women suffering from hypothyroidism—stepwise regression analysis.

**Statistically significant predictors**	***R*^**2**^ for the model**	**Adjusted *R*^**2**^ for the model**	**β**	**T**	***p***
**Dependent variable: depressive symptoms (B =** –**0.374; F = 15.170;** ***p*** **< 0.001)**					
Symptoms very visible to others	0.333	0.311	0.199	2.932	0.004
Negative attitude of other people to the disease			0.148	2.148	0.033
My illness is embarrassing			0.156	2.279	0.024
The treatment I get does not help me at all.			0.137	2.055	0.041
Compared to other people who have this illness, my symptoms are very severe			0.159	2.253	0.025
Lack of knowledge about the disease			0.138	2.168	0.031
**Dependent variable: anxiety (B = 3.427; F = 24.536;** ***p*** **< 0.001)**					
Compared to other people who have this illness, my symptoms are very severe	0.348	0.334	0.284	4.271	0.000
Lack of knowledge about the disease			0.244	4.007	0.000
Symptoms very visible to others			0.239	3.627	0.000
My illness is embarrassing			0.137	2.098	0.037
**Dependent variable: anger (B = 3.734; F = 7.615;** ***p*** **< 0.001)**					
Symptoms very visible to others	0.172	0.150	0.183	2.402	0.017
Age			−0.195	−2.813	0.005
Medical staff cannot influence the course of my illness			0.202	2.827	0.005
Significance of consequences			0.169	2.312	0.022
Relationship status			−0.134	−1.987	0.048

*Only significant predictors are included*.

The obtained regression models were statistically significant with relatively high percentages of variance explained for depressive symptoms (adjusted *R*^2^ = 0.31) and anxiety (adjusted *R*^2^ = 0.33). In both cases, beliefs pertaining to the social perceptions of the illness, such as the noticeability of symptoms, turned out to be important predictors. For anger, the amount of explained variance was comparatively smaller (adjusted *R*^2^ = 0.15) and, in this model, sociodemographic variables such as age (negative predictor) or relationship status (single or in a relationship) were found to be statistically significant predictors, in addition to illness-related beliefs.

## Discussion

### ED and Clinical Features of Hypothyroidism

Our results indicate that there is a relationship between the severity of major symptoms of hypothyroidism (i.e., intolerance of cold, swelling of the hands and/or feet and dryness of skin) and the severity of depressive, anxiety, and anger symptoms. It is worth noting that even though these symptoms are claimed to be the core subjective symptoms of hypothyroidism, they may not necessarily be directly caused by the pathophysiology related to hypothyroidism itself and may be due to other causes, such as physiological changes related to anxiety or other comorbidities. It should also be emphasized that the correlations we obtained do not provide evidence for a causative link, however, it is possible that emotional states such as depression or anxiety alter the threshold for perception of pain (40) or pruritus (41). In contrast, mixed findings are reported in the literature on the possible role of ED in modification of other cutaneous perceptions (42, 43). Our results may also mean that people with increased symptoms of hypothyroidism are prone to experience more pronounced ED as a psychological consequence of these symptoms, and, in fact the findings from our remaining analyses seem to confirm this conclusion.

This interpretation seems to be also corroborated by the lack of correlation between these symptoms and other indicators of disease severity, such as TSH, fT3, and fT4 levels and the dose of L-thyroxine used for treatment. In addition, no relationship was found between ED and hormone levels, duration of the disease, or doses of medication. These results are in line with the previous findings of other authors (44), according to which there is no relationship between thyroid hormones and the severity of ED (16). However, some studies found that the risk of occurrence of depressive symptoms or a diagnosis of affective disorder increased with the level of fT4 (45, 46).

### ED and Cognitive Illness Representation

Our study found a relationship between the severity of ED symptoms and beliefs which are components of cognitive illness representation. These beliefs were correlated with depressive symptoms, anxiety, and anger regardless of age, education, hormone levels, or time since diagnosis. In addition, the beliefs were better predictors of ED symptoms than the clinical variables. The analyses found, for example, that the subjective belief regarding the severity of symptoms compared to other people was a better predictor of symptoms of anxiety and depression than severity of physiological symptoms. In addition, a closer inspection of the contents of the illness-related beliefs which were found to best predict ED suggests that those pertaining to the anticipation of the social perceptions of hypothyroidism are most important—beliefs regarding the visibility of symptoms or embarrassment about the disease were found to be the most significant predictors of ED. These results are consistent with other studies indicating difficulties related to social functioning and health status in general (47, 48). Our findings are also in line with previous studies demonstrating the crucial role of IRBs in ED in other clinical samples, including patients with cancer (49, 50), acute myocardial infarction (51), diabetes (52), anorexia (53), and irritable bowel syndrome (54).

The results of our regression analyses, both hierarchical and stepwise, indicated that illness-related beliefs explain relatively high levels of the outcome variables (about 30% of the variance in depressive and anxiety symptoms and 16% in anger) as measured by HADS-M. It should be noted that the HADS-M measurement is not specifically limited to the illness situation and, therefore, the score on this scale may reflect ED in a response to a wide array of situations. In this context, the amounts of variance in these variables that our study shows can be accounted by illness-related beliefs should be viewed as relevant and high. It is of particular note that cognitive illness representation turned out to be a better predictor of ED than hormone levels, the duration of the disease, or the severity of symptoms. Furthermore, the increases of the variance in ED (*R*^2^-change; Table 4) explained by cognitive illness representation are much higher than those explained by either sociodemographic variables or clinical variables. Since illness-related beliefs were entered into the regression models in the last block, after the sociodemographic and clinical variables, as depicted in Table 4, their contribution to explain the variance in ED must be viewed as already cleared off the possible variance shared with the latter blocks of variables. These findings provide strong support for the hypothesis that ED in women with hypothyroidism is mediated by illness-related beliefs rather than a direct physiological consequence of altered hormone levels or other biological pathomechanisms.

### Limitations and Further Directions for Research

This study has some limitations. The design of the study contributed to the inevitable lack of control over the actual status of hypothyroidismin our participants—this information was provided by the respondents themselves. This obviously raises the question of reliability of such data when self-reported by patients. However, some studies suggest that data obtained in this manner are not necessarily unreliable. For instance, in a study conducted by D'Aloisio et al. (55), a diagnosis of breast cancer was later confirmed using real medical records in over 99% of women who self-reported this condition. On the other hand, thanks to the on-line survey method used in our study, it was possible to recruit a relatively large sample of patients at different stages of the disease from various locations in Poland which is an advantage in terms of the sample representativeness.

Also the lack of control of the diagnoses of particular conditions that were the cause of hypothyroidism might be considered as a limitation - in the study we focused on the symptoms of hypothyroidism - we did not control its possible causative pathophysiology, such as Hashimoto's disease or effects of radiation treatment. The aim of the study was to determine the role of psychological factors as contributing to ED, in the case of knowledge about the diagnosis of hypothyroidism. The lack of correlation between ED and medical factors may be due to the low variance of hormonal indicators and may be considered a limitation. However, our goal was to collect a group consistent in terms of hormonal indicators, and despite the fact that the patients were mostly euthyroid, we observed high occurrence rates of e.g., clinically significant symptoms of depression (20%) or anxiety (48.3%).

Another limitation is inherent to the cross-sectional nature of tour study, which precludes us from drawing conclusions on the direction of the relationships. Future research should use repeated measurement that would more effectively identify the factors that influence ED in this group of patients, with precise control of both cognitive variables and medical indicators.

Future research should also extend the model to assess the role of particular cognitive factors as well as social factors and protective personality and temperament factors (e.g., perceived social support or self-efficacy). According to previous findings (19), it is the diagnosis of thyroid disease itself that may be linked to depression, so further analyses should take into account the cognitive appraisal of the disease in addition to the cognitive representation of the disease and better control for the stages of the illness (e.g., at diagnosis, 1 year later, and 5 years later).

The results obtained in this study also have some practical implications. They can be used to develop psychological interventions (e.g., within the cognitive-behavioral approach, which focuses on changing specific beliefs), which could reduce the symptoms of ED. These results may also be useful for physicians and public health professionals. In the case of doctors, they can be used to adequately educate patients, provide information consistent with medical knowledge, and thus form a different representation of the disease in accordance with the latest knowledge. Additionally, the clinician should remember that in patients treated with L-thyroxine, with target TSH, some ailments may be due to psychological factors (e.g., illness belief), and not the incorrect dose of the hormone. In such patients, psychological consultation might be considered as optimal solution reducing patients ED. In the case of public health specialists, the results of the study can be an inspiration and theoretical basis for various campaigns aimed at beliefs and expanding knowledge about the disease, which could ultimately contradict the improvement of the psychological well-being of women struggling with this health problem.

In summary, the data collected in our study allow us to conclude that psychological factors, and in particular—illness representation—play a greater role than clinical ones (severity of symptoms, duration of disease or hormone level) in the development of depression, anxiety, and anger in patients with hypothyroidism. These results are consistent with the analyses of other researchers, emphasizing that awareness of the diagnosis (and hence the associated cognitive factors) plays a greater role than biological factors (hormone levels, disease characteristics) in the etiology of ED symptoms among patients with this thyroid disease. In addition, our results may have practical implications—psychosocial interventions targeting personal beliefs about the nature of the disease and its social aspects may prove to be an effective way to reduce ED symptoms.

## Data Availability Statement

For raw data supporting conclusions for this article, please contact the authors. The database contains medical data of our patients and we cannot make this part public.

## Ethics Statement

The studies involving human participants were reviewed and approved by University of Economics and Human Sciences in Warsaw Ethical Board. The patients/participants provided their written informed consent to participate in this study.

## Author Contributions

DP: conceptualization, methodology, formal analysis, investigation, resources, data curation, writing - original draft, writing - review & editing, visualization, supervision, and project administration. KW-P: conceptualization, methodology, investigation, resources, data curation, writing - original draft, writing - review & editing, visualization, supervision, project administration, and funding acquisition. KJ: conceptualization, methodology, writing - original draft, and writing - review & editing. EP and MW: writing - original draft and writing - review & editing. All authors contributed to the article and approved the submitted version.

## Conflict of Interest

The authors declare that the research was conducted in the absence of any commercial or financial relationships that could be construed as a potential conflict of interest.

## Abbreviations

TSH: Thyroid-Stimulating Hormone
fT4: free thyroxine
fT3: free triiodothyronine
NHANES III: (United States') National Health and Nutrition Examination Survey
L-thyroxine: Levothyroxine
IRBQ: Illness-Related Beliefs Questionnaire
HADS–M: Hospital Anxiety and Depression Scale-Modified
M: mean
SD: standard deviation.
