# Supplementary material for: The Role of Illness-Related Beliefs in Depressive, Anxiety, and Anger Symptoms: An On-line Survey in Women With Hypothyroidism
Source: Front Psychiatry. 2021 Apr 22;12:614361. doi: 10.3389/fpsyt.2021.614361 (PMC8100212; doi:10.3389/fpsyt.2021.614361)
Supplement: Supplementary file 1 [file Data_Sheet_1.docx]

**Illness-Related Belief Questionnaire**

Version P-13

**Instructions**On the other side of this sheet, you will find 13 pairs of statements concerning **your** illness. Each pair presents two opposing statements about a certain part of the illness, for example:

| The symptoms of my illness are not painful at all. | 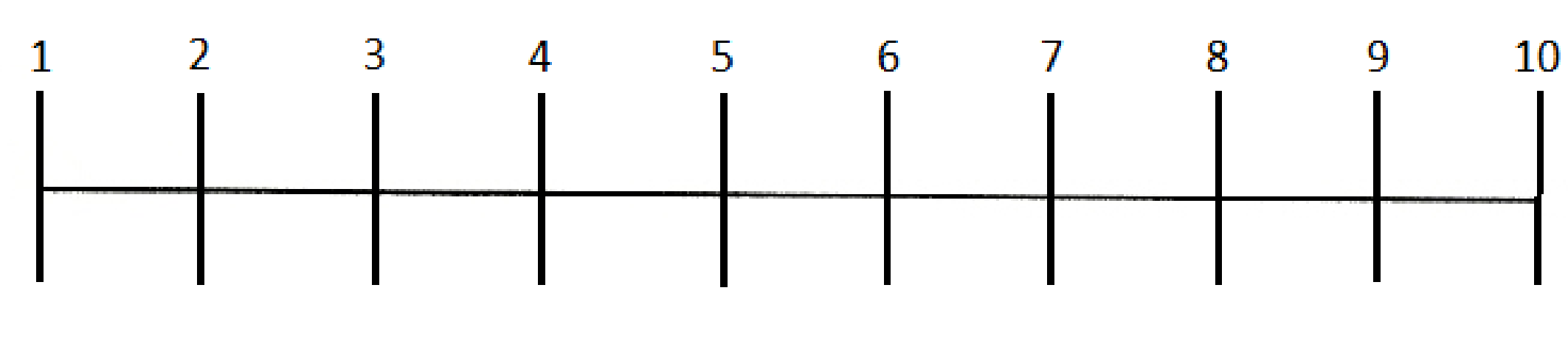 | The symptoms of my illness are very painful. |
| --- | --- | --- |

The numbers between these statements indicate how much you agree with the statement on the left or on the right:

closer to 1: you agree more with the statement on the left

closer to 10: you agree more with the statement on the right

**Please read each pair of statements on the other side of the sheet carefully.**

**For each pair, draw a circle around the number that best corresponds to the belief you agree with.**

*Examples:*

| The symptoms of my illness are not painful at all. | 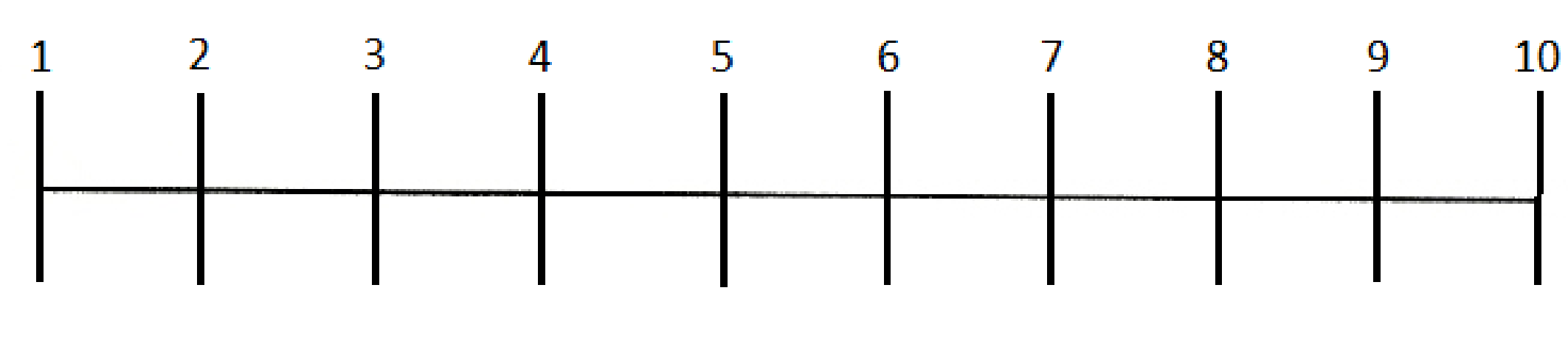 | The symptoms of my illness are very painful. |
| --- | --- | --- |

In this example, the answer means that the symptoms of your illness are not very painful.

| The symptoms of my illness are not painful at all. | 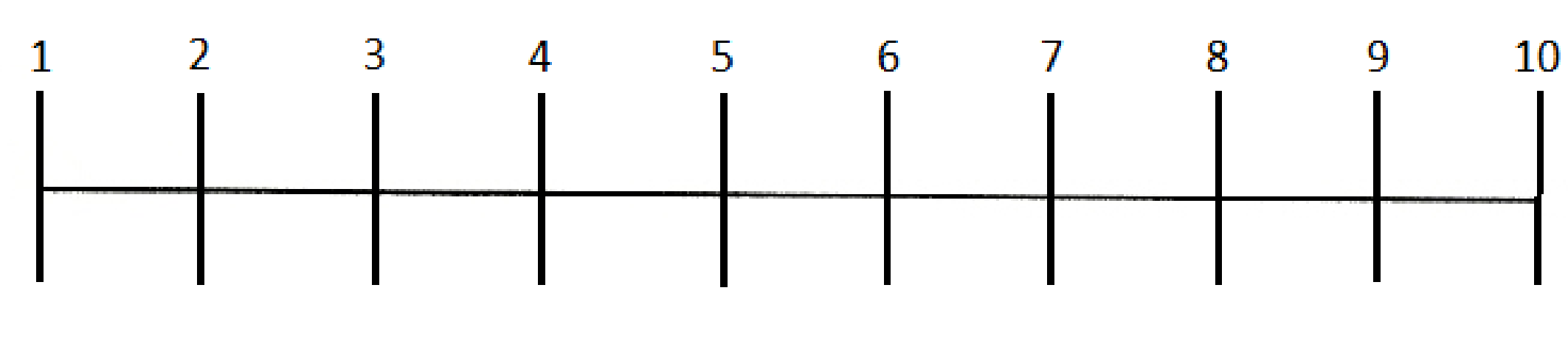 | The symptoms of my illness are very painful. |
| --- | --- | --- |

Here, the answer means that the symptoms of your illness are moderately painful.

| The symptoms of my illness are not painful at all. |  | The symptoms of my illness are very painful. |
| --- | --- | --- |

In the last example, the answer means that the symptoms of your illness are very painful.

My illness: …………………………………………………….

| My illness will last for a short time. | 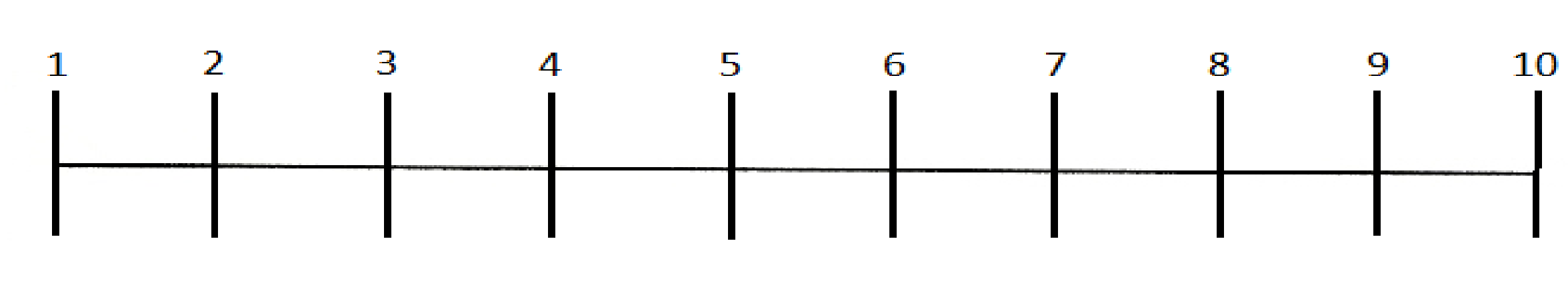 | My illness will last for a very long time. |
| --- | --- | --- |
| This illness will not have a significant impact on my life. | 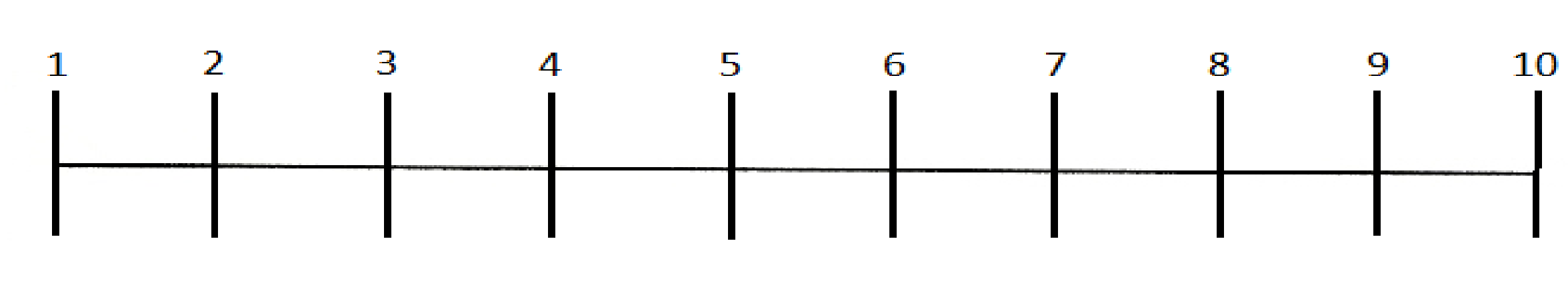 | This illness will have a very significant impact on my life. |
| My condition will improve. | 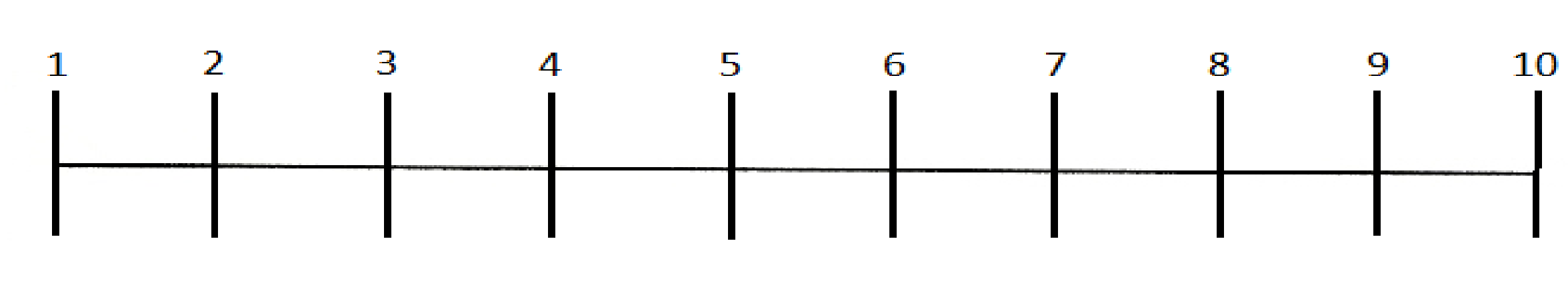 | My condition will worsen. |
| The symptoms of my illness are not visible to others. | 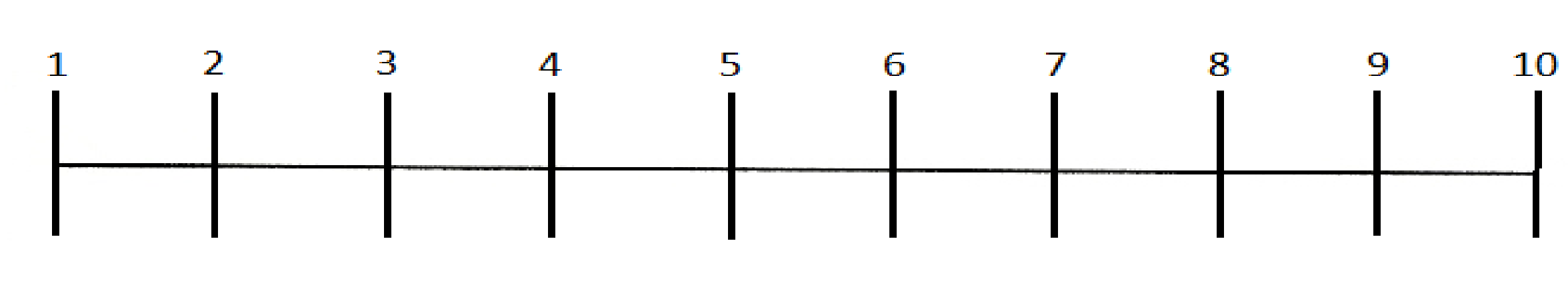 | The symptoms of my illness are very visible to others. |
| I am able to fully predict the course of my illness. | 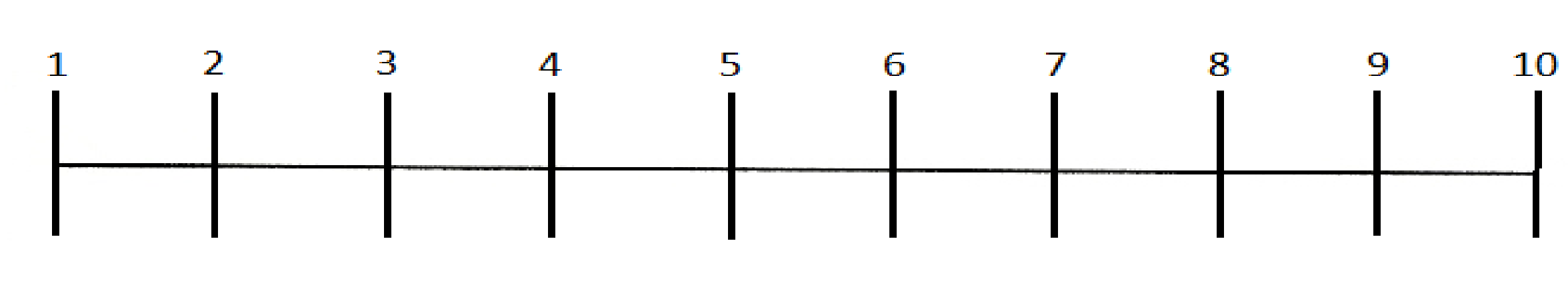 | I cannot predict the course of my illness at all. |
| I know a lot about my illness. | 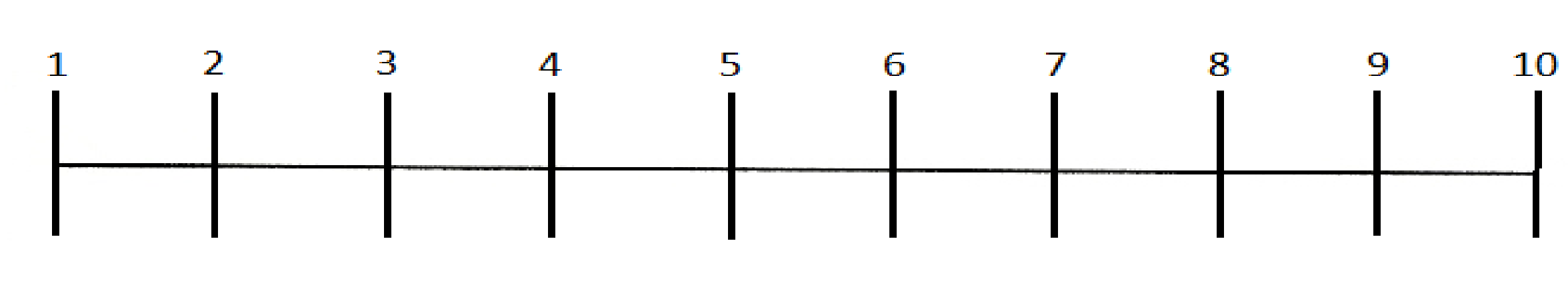 | I know almost nothing about my illness. |
| I had no control over becoming ill or not. | 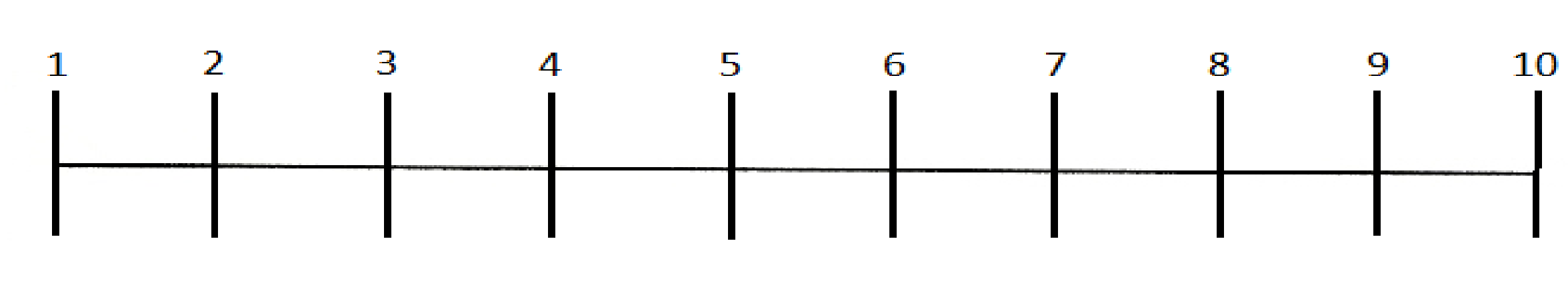 | The things I did caused me to become ill. |
| The things I do can influence the course of my illness. | 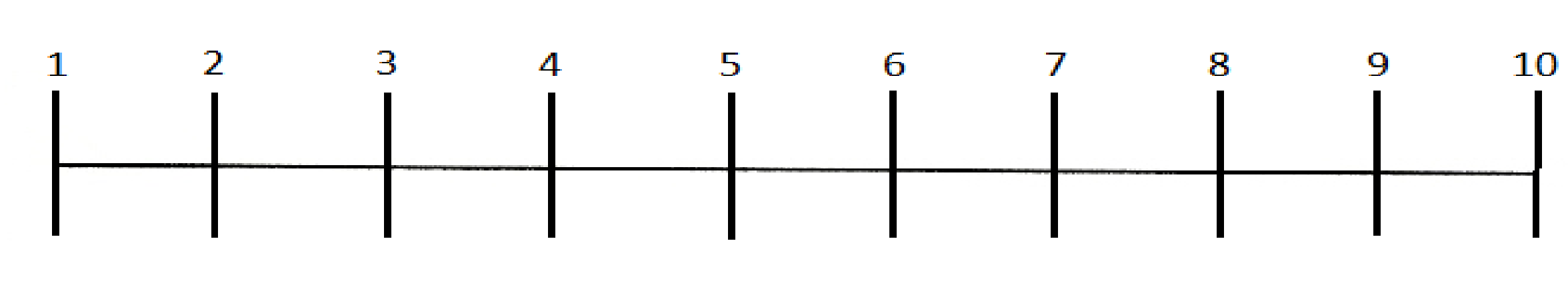 | The things I do have no effect on the course of my illness. |
| The medical staff can influence the course of my illness. | 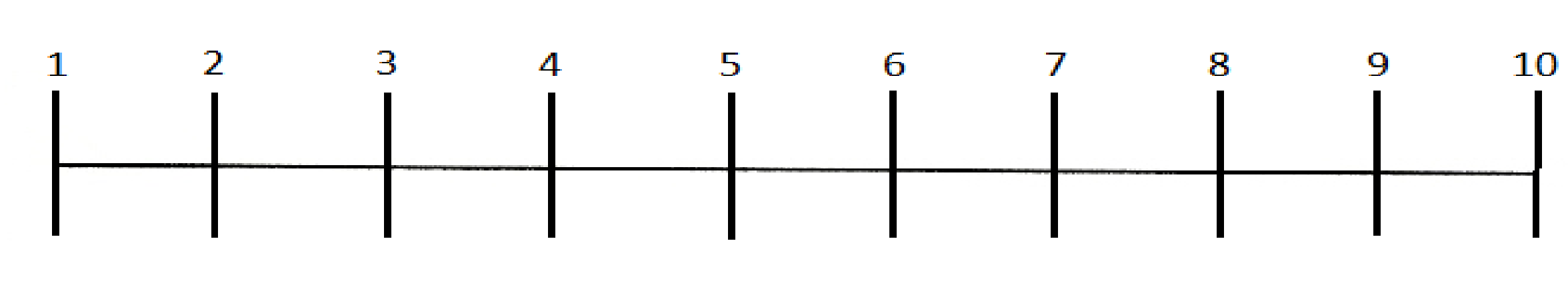 | The medical staff cannot influence the course of my illness. |
| The treatment I get is very effective. | 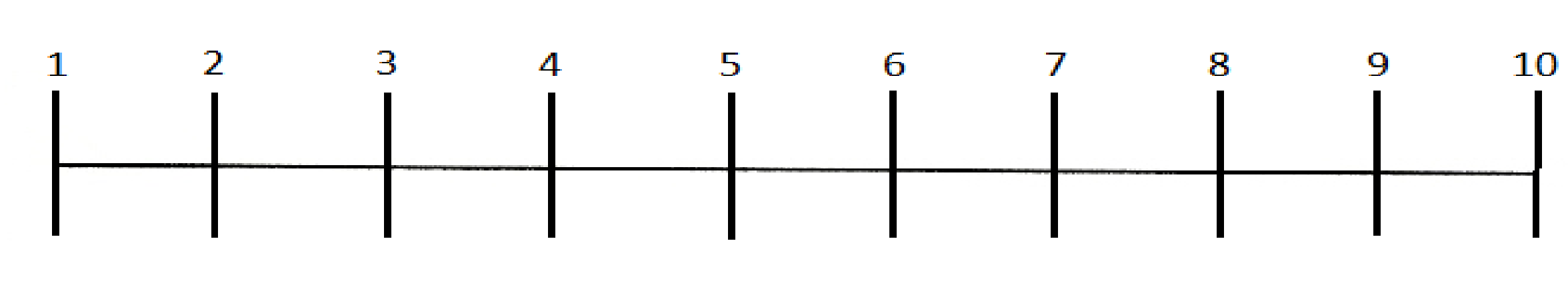 | The treatment I get does not help me at all. |
| In general, others would regard me positively as a person who has this illness. | 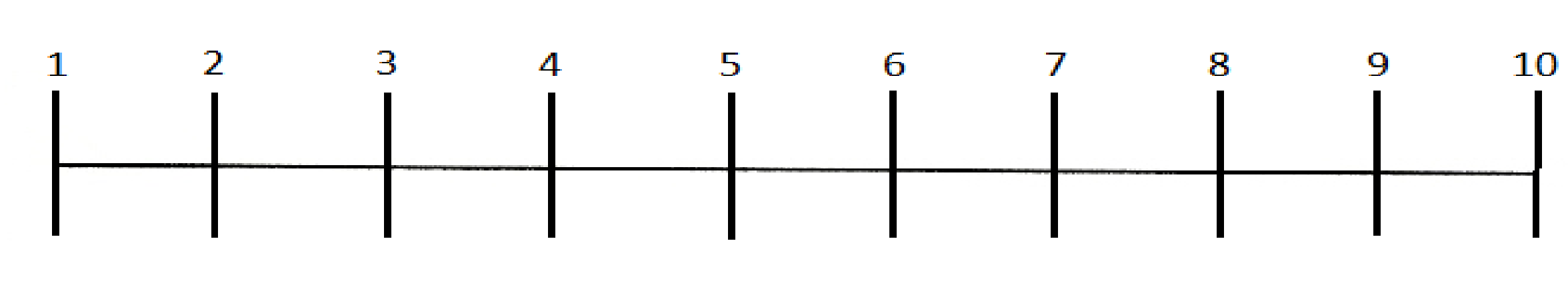 | In general, others would regard me negatively due to the fact I have this illness. |
| I believe my illness is not embarrassing. | 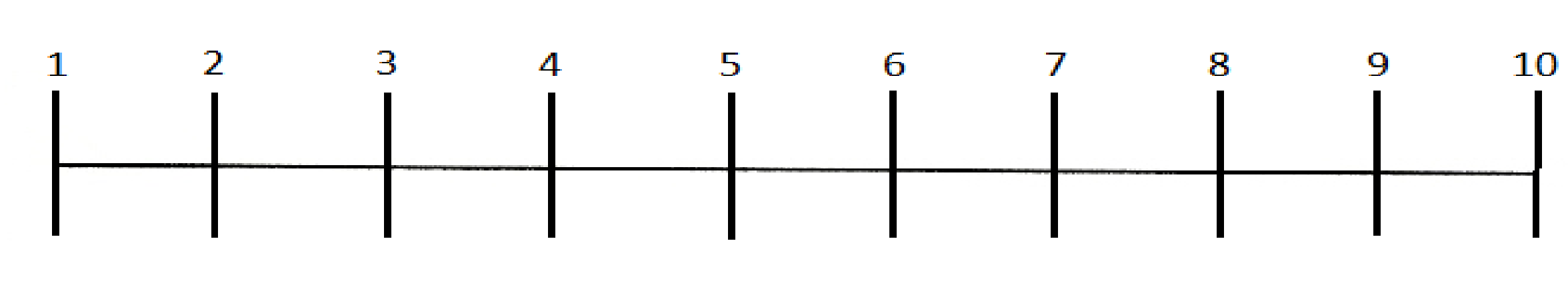 | I believe my illness is embarrassing. |
| Compared to other people who have this illness, my symptoms are very mild. | 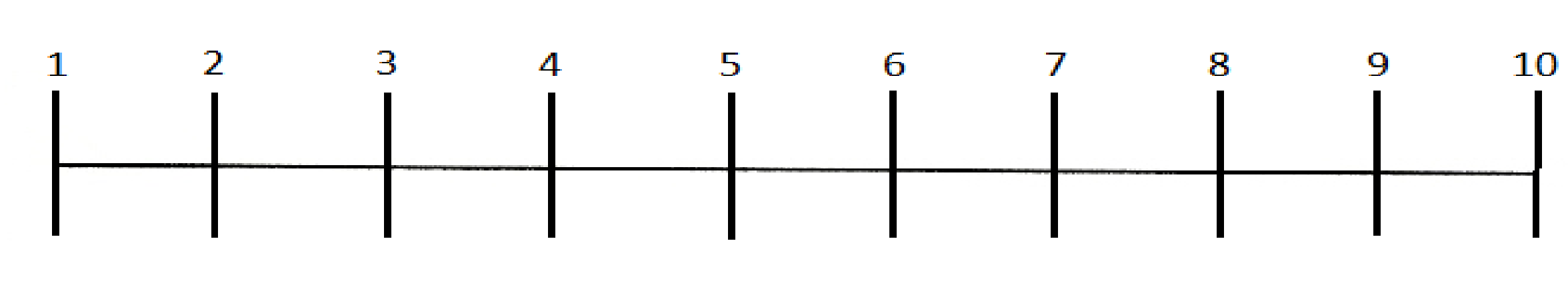 | Compared to other people who have this illness, my symptoms are very severe. |
